# Supplementary material for: Chromothripsis during telomere crisis is independent of NHEJ, and consistent with a replicative origin
Source: Genome Res. 2019 May;29(5):737–49. doi: 10.1101/gr.240705.118 (PMC6499312; doi:10.1101/gr.240705.118)
Supplement: Supplemental Material [file supp_gr.240705.118_Supplemental_file_1.zip › contigs/annotated_contigs/DB37/contig.2.DB37_length_706_mean_cov_7.22379603399.docx]

**DB37_length_706_mean_cov_7.22379603399**

```
TCAGATTTAGATGTTTTTCCAATGGCCAGGCTCACAAAATAATCCAGACACCAAACTTGTAAGCTGGCTAAAAGCGGCAAGGAATGATT
 >chr6:45705650-45706008 + E=1e-200
CTTGGCTTATCTCCCACTGGATTTTACTTACTGGGCTATTCAAGTGGATTATTTCATTCAAGGTAGAAAGCCCTCCACACGGAGGGCTG

GACAAATTCCCAGCACACCCAAGCTATTAGAAGGCCTTTCAGGGATGACAAGAATATGTTAGCTTCATTTCTTCTCTCTCACAAGAGAC

TCTTCCCTCCCACGAAAAGACTGAAACAATAGATTTACCCAAAGATGGCCGCTCTGAGGGCCTGCCCTTGATGAGGTTAAAGGAAGTGG

CT|TACTTGCCAGGGTCTAGGAGTTGTTTTGGGTCATCTCTAAAAGTCCTCTAGGGATCTAAAATCTATTTGTAATCTGGATACAAAAC
 >chr6:45457904-45458254 + E=8e-199
AGTAATGGTGGGGAAAACAACCATTAACAATGAATTTCTCTAATTTTTCCTGTTCATTTTTCTCCTAGGTCTAGTCCTTCTGTTATAGC

AGATGCCCAAAAACTTGCACTGGCAGGAAATAGGGTGGTGGGTGCATTTCAGTTAATGGGCCTAGTTGGAACACGTTTGGCCGTGGAGA

CAGCTACACTCATATTACCAAGCCTTGGCTTCCTATCTGTTTTCTTTGGGCTGGGAAGGCAATGAAAAAGGAGACAGGAAAATA
```
